# A Singular Case of Diabetes, Consisting Entirely in the Quality of the Urine; with an Inquiry into the Different Theories of That Disease

**Published:** 1788

**Authors:** Thomas Cawley

**Affiliations:** late chief Surgeon to the Forces in Jamaica


### IV. *A ſingular Caſe of Diabetes, conſiſting entirely in the Quality of the Urine; with an Inquiry into the different Theories of that Diſeaſe. By* Thomas Cawley, *M. D. late chief Surgeon to the Forces in Jamaica.*

ALLEN HOLFORD, Eſq., aged thirty-four years, ſtrong, healthy, and corpulent, accuſtomed to free living and ſtrong corporeal exertions in the purſuit of country amuſements, in December, 1787, was ſeized with diabetes; but the cauſe of the great degree of emaciation and debility which gradually came on was not diſcovered until March 20th, 1788, at which time his urine was found to be ſweet, fermentable with yeaſt, and two pounds, on evaporation, yielded about five or ſix ounces of ſweet black extract, exactly reſembling that preparation of melaſſes made by confectioners for children, and vulgarly called *coverlid*.

Within the above-mentioned period the quantity of urine evacuated was never obſerved to exceed what is uſual in health, or to be diſproportioned to the ingeſta, though the ſtate of it had been frequently inquired into, and even the quantity of liquids drank and voided meaſured. For theſe reaſons the quality of it was not ſuſ-

pected pected until it became inconceivable, considering the quantity of aliment taken in, how such a degree of exhaustion could ensue, unless the body was drained by the quality of what was rejected as apparently excrementitious.

Variety of medicines, the usual consequence of inefficacy and despair, were successively administered. Decoction of bark with vitriolic acid and alum, with astringents and aromatics, with chalybeates, with sacc. saturni and opium, and with cantharides, together with cold bathing in salt water, were the principal means used, and at first had a very good effect; but soon afterwards every medicine disagreed with the stomach, and the patient gradually sunk and died on the 18th of June.

The disease was at first attended with severe pain in the rectum, caused by piles, and all the while a considerable degree of costiveness, the usual cause of hæmorrhoidal affections, prevailed. For some time before his death slight hectic symptoms appeared: his thirst became intolerable; his mouth and fauces very clammy; his tongue deeply chapped; his skin dry and scaly; and his appetite, which at first was tolerable, gradually diminished, and latterly was changed into an aversion even of a sight of solid food.

food. His only ſupport, therefore, in this ſtage of the diſeaſe, was derived from a plentiful ſupply of nutritious liquids.

About this time, when he voided urine, he uſually applied his hands to the hypochondria, and expreſſed a ſenſation of ſinking, as if the urine came from thoſe parts.

Within the laſt three days of his exiſtence the quantity of urine was conſiderably increaſed, the power of retention much diminiſhed, and his right arm was frequently agitated with convulſive motions for a few minutes, and then became ſteady. Delirium and convulſions cloſed the ſcene.

Notwithſtanding this progreſſive increaſe of fatal ſymptoms, the only apparent cauſe, the ſaccharine matter in the urine, daily decreaſed in proportion, and latterly two pounds yielded only an ounce and a half, whilſt the quantity of urine evacuated ſeldom exceeded four or five pounds in twenty-four hours, and had changed from a very light ſtraw colour to one deeper and more natural.

### *Appearances on Diſſection.*

The kidnies were of the uſual ſize, but appeared to me to be rather paler and ſofter than what what is natural; when opened longitudinally through the pelvis, nothing preternatural was discovered.

The liver was much wasted. It was externally of an ash colour, or nearly like pipe clay, and its consistence was very plastic, or like an œdematous tumour, which might be moulded, like dough, into any shape. It was perfectly free from any scirrhous or steatomatous tumours taken notice of by Dr. Mead, and, when cut into, exhibited its usual colour.

The gall bladder contained its accustomed quantity of bile, and adhered to the mesocolon.

The pancreas was full of calculi, which were firmly impacted in its substance. They were of various sizes, not exceeding that of a pea, white, and made up of a number of lesser ones, which made their surface rough, like mulberry stones; and in all respects they appeared analogous to the calculi which we sometimes meet with in the salivary ducts. The right extremity of the pancreas was very hard, and appeared to be scirrhous.

No other marks of disease could be discovered in the abdomen, and the contents of the thorax were perfectly sound.

*Obser-*

### *Obſervations and Experiments on the Urine and Extract.*

There was no oilineſs on the ſurface of the urine; when freſh, it had a very faint ſweet odour; and when kept two or three hours in a cloſe warm place, it began to ſmell ſour. During evaporation a ſlight urinous odour was diffuſed; but this was ſcarcely perceptible in the extract, and in ſome parcels of it not diſcernible.

Experiment I. A ſmall quantity of urine, ſet by in a phial, ſpontaneouſly entered into the vinous, and then into the acetous fermentation, diſcharging a great quantity of mephitic gas. A white cloud formed in the center, which gradually fell to the bottom in the form of a white precipitate. In ſhort, the whole of this experiment correſponded with Dr. Dobſon's *.

Experiment II. Vitriolic acid poured into the urine cauſed no change; neither did fixed alkali, when added to it, excite any pungent odour. This proves that the urine contained very little or no ammoniacal ſalt, as the fixed alkali, by decompoſing it, and ſetting the vo-

* Medical Obſervations and Inquiries, Vol. V. p. 303.

latile latile alkali at liberty, would have excited a pungent ſmell.

Experiment III. A ſmall quantity of the extract put into water diſſolved very rapidly.

Experiment IV. A ſmall quantity of the extract put into ſpirit of wine neither diſſolved nor communicated any colour to it, but immediately became very hard and brittle.

It appears, by the laſt experiments, that the extract conſiſts of ſugar united with gummous or coagulable matter, all which ought to remain in the body for its ſupport, and that little of what is excrementitious paſſed through the kidnies but ſuperabundant water, the vehicle of this nutritious matter.

Healthy urine yields, on evaporation, more or leſs of coarſe black or brown extract; but this extract has a ſtrong urinous ſmell, deliquates when expoſed to the atmoſphere, and is ſoluble in ſpirit of wine, being in its nature ſaline and ſaponaceous, and entirely excrementitious *.

As the analyſis of urine does not ſeem to have been properly attended to by writers on the diabetes, I think it neceſſary here to enter more fully into it than has hitherto been done. This

* Vide Journal de Medecine, Nov. 1773 & Avril 1777.

 I ſhall

I ſhall do in the words of a late celebrated writer *.

" A meſure que l'urine s'evapore, elle prend " une couleur de plus en plus brune & foncè, " par le rapprochement de la partie ſavonneuſe " extractive qu'elle contient. Les premiers " criſtaux qu'on obtient, ſont l'eſpece particu- " liere de ſel connu par les chimiſtes ſous les " noms de *ſel natif ou eſſentiel de l'urine, ſel fu-* " *ſible de l'urine, ſel phoſporique, ſel microcoſ-* " *mique.* C'eſt celui qui contient l'acide pro- " pre a faire le phoſphore. Il y a une partie de " ce ſel qui eſt a baſe d'alkali volatil, & qui eſt " par conſequent de nature ammoniacale; l'au- " tre partie eſt a baſe d'alkali fixe mineral.

" En continuant l'evaporation & le refroi- " diſſement alternatifs, on retire ſucceſſivement " de l'urine les autres ſels moins criſtalliſables " qu'elle peut contenir, mais principalement le " *ſel commun*, ou le ſel febrifuge de *Sylvius*, dont " elle eſt toujours abondamment chargée. On " retrouve auſſi tous les ſels neutres qu'ils ont " pris, ſoit par la voie des alimens, ſoit autre- " ment."

* Macquer. Dictionnaire de Chimie. 4to, Tom. II, p. 645.

From

From this analyſis it appears that healthy urine contains a variety of ſaline matter, the principal of which are the following: — Phoſphoric ſalt, or ſalt compoſed of phoſphoric acid and vegetable, mineral, or volatile alkali; common ſalt; and the febrifuge ſalt of Sylvius, or ſalt compoſed of marine acid and vegetable alkali.— As phoſphoric acid and volatile alkali are generated in the body, we may eaſily ſuppoſe the following chemical decompoſitions and attractions to take place: — The common ſalt taken in with the aliment is decompoſed by the phoſphoric acid, which unites with its alkaline baſis, and forms one ſpecies of the fuſible ſalt of urine: the marine acid, now at liberty, unites with the vegetable alkali taken in with our aliment, and forms the ſalt of Sylvius; and the ſuperabundant phoſphoric acid, uniting with the volatile alkali, forms the ammoniacal phoſphoric ſalt. The quantity of theſe ſalts muſt be variouſly proportioned, according to the quantity of aliment taken in, the quality of it, and the intervals of repletion.

Hence it appears probable that the want of ſaline matter, or ammoniacal ſalt, ſo much talked of, in diabetic urine, proceeds from a deficiency of phoſphoric acid and volatile alkali, without which the faline particles taken in with our aliment cannot be decompofed, or form any new combinations, but muft be ejected by the excretive powers as they entered.

The *acidum perlatum*, which has been difco- vered in microcofmic falt, is too little known to require any attention at prefent *.

### *Inquiry into the different Theories of this Difeafe.*

The confideration of the above cafe naturally leads to an inquiry into the different theories of this difeafe. Is it a defect of affimilation, a difeafe of the liver, or an affection of the kid- nies?

As to affimilation, the antecedents of the dif- eafe point out no defect in digeftion. It has frequently attacked perfons in the vigour of life, and has ufually been attended in all its ftages with a voracious appetite; from which it may be inferred that digeftion has not only been pro- perly performed, and the chyle conveyed into the circulation in a ftate fit for nutrition, but experiment confirms it. The ferum of blood

* Vide Bergman on Elective Attractions.

taken taken from the arm had no preternatural ſweetneſs *.

If want of aſſimilation, and its ſuppoſed conſequence, are the effects of a weakened ſtate of the animal functions, why is not diabetes the uſual concomitant of that ſtate?

That the cauſe of diabetes and quality of the urine have long been ſubjects of ſpeculation, and that the idea of defect in the aſſimilatory powers is not new, will appear from the following quotations:

" Ad renes pertinere is affetus videtur, quem

" alii hydropem matellæ, alii urinæ profluvium,

" alii diabetem, alii Διψακον appellant. Equi-

" dem cum hactenus bis duntaxat videre potui,

" ſupra modum ſitientibus infirmis, atque ſu-

" binde bibentibus. Quare exuberanter quo-

" que reddunt, id quod biberunt, *eo a ſua qua-*

" *litate non mutato.*"—*Galen*, l. 5. de loc. affect. cap. 3.

" Cauſam vero hujus affectionis reddere dif-

" ficillimum, & pauci autores inveniuntur, qui

" in ea reddenda inter ſe conveniunt. Nos in

" re obſcura, ſalvo cujuſque judicio, ſtatuimus,

" proximam hujus mali cauſam eſſe facultatem

* Home's Clinical Experiments, page 308.

" renum

" renum retentricem læsam, & quidem ab urinæ " vel copia, vel *qualitate*."—*Sennert*. op. Folio. Lugdun. 1650. Tom. II. p. 1094.

In the same chapter from which the preceding quotation is taken is the following question: — " An potus immutatus planè per urinam in dia- " bete reddatur?" — Galen, Alex. Trallian, Aetius, Amatus Lusitanus, and Trincavellius, say it is not changed; and the latter observes, that in one case he found it " servans eundem " calorem, consistentiam, saporem atque odo- " rem." — *Ibid*. p. 1095.

Dodonæus, J. Baptista Sylvaticus, and others, having taught a contrary doctrine, Sennertus, in giving his own opinion, attempts to reconcile both. — " Ideoque in diabete non solum ad po- " tum, sed etiam alia respiciendum. Sunt " enim primò diabetæ quidem gradus. In " principio enim, cum vires nondum dejectæ " sunt, & vis alteratrix, nondum extremè labo- " rat, non mirum est, si potus aliquomodo mute- " tur; temporis vero progressu, ubi vis altera- " trix magis labefactatur, potus plane immuta- " tus excernitur. Deinde potus etiam alius mu- " tatur facilius, alius difficilius. Aquam, cum " parum mutari possit, non mirum est, eodem " colore & reliquis accidentibus non mutatis,

" excerni:

" excerni : alii vero potus, qui magis compositi " sunt, non ita facile transeunt, quin aliquam " mutationem accipiant. Præterea id, quod in " diabete excernitur, non saltem potus est, sed " sæpe etiam accedit corporis colliquatio, unde " plus urinæ emittitur, quam potûs assumptum " est."—*Ibid.* p. 1095.

Then comes the following question: "Quænam diabetæ causa sit?"—Answer. "Vulgata " quidem, & quam plerique sequuntur, sen" tentia est, proximam hujus mali causam esse " renum intemperiem calidam, ob quam illi " serum copiosius e venis attrahant, quodcum " ob imbecillitatem & copiam retinere non pos" sint, venas rursum a jecore, hoc ex intestinis " & ventriculo trahere, unde orificium ventri" culi vellicetur, ac sitis excitetur, ob quam " assumtus potus mox a venis & renibus attraha" tur, atque iterum ad vesicam mittatur."—*Ibid.*

J. Baptista Sylvaticus, after Galen, Aretæus, and Actuarius, having delivered the following doctrine—"Insignem calidam intemperiem in " hepate & toto venoso genere succensam, san" guinem fundere, ejusque portionem aliquam " in serum mutare."—Sennertus farther observes, " His autoribus posterioribus assentimur in eo,

" quod

"quod non tam in renibus quam aliis partibus "causa diabetis quærenda sit. Sit enim, de "quo tamen, ut ex superioribus patet, non im- "merito dubitatur, quod renes fortiter attrahant: "tamen nisi serum adsit, id attrahere non pos- "sunt; & renum fortis attractio, seri seu urinæ "copiam jam præsupponit."—*Ibid.*

"Quapropter diabeten sanguinis potius & "immediatius quam renum affectionem esse, & "originem suam inde sumere credimus, qua- "tenus cruoris massa velut deliquescit, & in "serositatem copiosè nimis funditur: quod e- "quidem ex urinæ quantitate ita in immensum "aucta, quæ non nisi a sanguinis deliquio, & "consumptione procedat, facile constat.... "Itaque.... opinari ducor sanguinis crasin sive "mistionem ita laxari, & quadantenus dissolvi, "ut particulæ aquosæ a crassioribus contineri "nequeant, quin illæ harum amplexionibus "cito elapsæ, & salinis imbutæ, per vias re- "num maxime patentes excurrant."—*Willis*, Pharm. Ration. p. 105.

"I believe the chief and most frequent cause "of diabetes consists in the too-much dissolved "and lax mixture of the blood."—*Bonetus*'s Guide. Folio translation. Book iv.

"For

"For their blood being by this means ſo im-

"poveriſhed as to be utterly unable to aſſimilate

"the juices received into the maſs, they paſs off

"crude and undigeſted by the urinary paſſages."

—*Swan's Tranſlation of Sydenham*, p. 313.

The diſcovery of the circulation of the blood naturally deſtroyed the theory of attraction by a ſuppoſed *calida intemperies*; and the diſcovery of the quality of diabetic urine by Willis, ſettled all diſputes on that head: but the cauſe of the diſeaſe, notwithſtanding thoſe great diſcoveries, ſeems ſtill to remain as unſettled as ever.

A late very celebrated writer* has pitched upon the liver for the ſeat of diabetes. He ſays he always found a ſteatomatous collection in it; to which he attributes a vitiated ſecretion of bile, deficient of ſaline matter to properly mix and aſſimilate the fluids.

This theory, as it is nearly allied to the circumſtances of Mr. Holford's caſe, appears plauſible; but it muſt be obſerved that the ſtate of the liver, deſcribed by Dr. Mead, differs eſſentially from that of the caſe before us: this, however, is not a ſufficient objection, as different ſtates of the liver may be ſuppoſed to produce a mor-

* Mead. Eſſay on Poiſons, page 28.

bid ſtate of bile, ſimilar, or equally unfit for the purpoſes of aſſimilation. It is neceſſary, therefore, to oppoſe authority to authority; and here another very eminent author aſſures us, that though the liver has ſometimes been found diſeaſed, yet this concurrenee does not often take place.—" In twenty inſtances," ſays he, " which " I have ſeen, there was not, in any one of " them, any evident affection of the liver *."

To this authority, excluſive of the negative proofs contained in the following diſſections, may be added the teſtimony of Dr. Home, who ſays, " the liver was natural †." May we not, therefore, conſider the diſeaſe of the liver as a complication in the caſe of Mr. Holford? and may not the ſame be ſaid of the calculous ſtate of the pancreas?

Unfortunately we have few diſſections of this diſeaſe to refer to; nevertheleſs what we have, excepting Dr. Mead's general aſſertion, expreſſly deſcribe ſuch an affection of the kidnies as we might, *a priori*, expect to find.

" Anno 1590, Filia Præſidis Hollandiæ 18 " annorum aliquot ante obitum annos diabete

* Cullen. Firſt Lines, Vol. IV. page 89.

† Clinical Experiments, page 311.

" labo-

"laborabat.... Renes huic non absumpti, verum flaccidiores solito, figura cineritia non impense rubra."—*Petrus Pawius* *, Ob. An. 2.

"Aperto cadavere ren sinister inventus est lapide obsessus exiguo: ren in magnam molem undique increverat, adæquabat renem bubulum magnitudine: paulum saniei in eo erat: dexter adeo parvus erat ut fere reperiri non potuerit; macruerat multum."—*Ballonius* †, eph. & epid. l. 2. p. 152.

"In Nob. N. a febre ardente extincto, pulmo niger & admodum tumidus repertus est; in utroque rene duo magni calculi: hic copiosiores justo fundebat urinas, aquæ simillimas, sitimque intolerabilem patiebatur, ut quæ nullo potu sedari poterat ‡."

"Ren sinister lapide angulos obtusos habente obsessus est, in ureteris principium implantatus..... Ren alter lapide non obsessus, justo minor erat, & pene collapsus. Nullum commemorabile vitium quod sub obtutum caderet."—*Ballonius* §, eph. & epid. l. 2. p. 183.

* Vide Sepulchretum Boneti, Lib. iii. sect. xxvi. ob. 1.

† Ibid. ob. 2.

‡ Ibid. ob. 3.

§ Ibid. ob. 5.

" Hic enim intra decem horarum decursum " ultra duodecim urinæ mensuras, incredibile " dictu, excreverat: & post aliquod tempus, " accedente alio graviori morbo defunctus, at- " que secatus, ureterem dextrum, insigniter & " farciminis instar expansum, quin ejusdem la- " teris renem in molem sinistro duplo majorem " elevatum ostendit *."

" Remotis intestinis, &c. in oculos mihi in- " currit ureter dexter mirum in modum distor- " tus, atque hic illic multum expansus, ut in- " testinum ratione crassitie representaret. Pel- " vis quoque adeo erat distenta ut malum au- " rantium mediocre facillime, & citra difficul- " tatem admitteret. Parenchymate omnimodo " consumto, nil præter membranosas partes, " perquam induratas superstes videbatur †."

" Vir quidam in ætatis flore, diu atrocibus " nephriticis doloribus vexatus, renisque ab- " scessum passus in diabeten incidit. Singulis " septimanis dolium dimidium cerevisiæ ingur- " gitare difficile illi non fuit. Post mortem. . . " viscera satis bene constituta, exceptis renibus " & ureteribus, conspexi; uterque enim ren ex

* Hoffmanni Consult. & Resp. Med. Casus 85.

† Ruyschii Dilucid. Valv. obs. 13.

" parte

"parte consumtus erat, præsertim dexter; cu-

"jus substantiâ planè consumtâ, ejus membra-

"nas summopere incrassatas & contractas, pel-

"visque capacitatem adæquantes vidi *."

"On examining the kidnies, the left was "larger than natural, and its substance softer. "There was no uncommon appearance in the "right kidney, except a greater degree of "softness. The substance of both kidnies had "a sour odour †."—Dr. Monro observes on the above case, that "both kidnies seemed to be of "a large size, were of a remarkably pale co-"lour, and felt rather softer than common ‡."

Morgagni has been quoted on this subject; but the case ep. 41. art. 13. confirms nothing, the kidnies not having been examined; and ep. 42. art. 43. he says, "Nec Valsalva, nec ego "quenquam ex diabete mortuum dissecui-"mus."

From the proofs above adduced, extracted from the most respectable writers, it appears that the kidnies have invariably been found considerably diseased; but as it has been fashionable, in consequence of a revival of the crude

* Ruyschii Obs. Anat. Med. Centuria, ob. 13.

† Home. Clinical Experiments, page 310.

‡ Ibid.

system

ſyſtem of the laſt century, to call this the effect, and not the cauſe, it is neceſſary to inquire more particularly into the circumſtances of the diſeaſe; the remote or occaſional cauſes of it; and the method of cure which has now and then ſucceeded.

An increaſed diſcharge of urine, excepting in the caſe of Mr. Holford, has always been the firſt ſymptom of diabetes; the other ſymptoms have been conſequential and in proportion. Can an imperfect digeſtion or aſſimilation be ſuppoſed capable of ſtimulating the kidnies to *excrete* five or ſix times the uſual quantity?

Chyle is at all times mixed with the blood, the blood veſſels being the vehicles of it, or the organs of its diſtribution. If diabetes be not a diſeaſe of the kidnies, why do they not permit the nutritive, chylous, or ſaccharine matter to paſs at all times? For it cannot be denied that ſuch ſaccharine matter is perpetually conveyed, mixed with the blood, by the emulgent arteries, and preſented, along with the excrementitious matter, to the excretory veſſels of the kidnies.

The principal argument againſt diabetes being an affection of the kidnies is this — " Sugar is " found in diabetic urine. Sweet chyle is the " firſt product of the ſtomachic and inteſtinal " digeſtion;

" digeſtion ; as chyle in the thoracic duct, and

" milk, which is a ſpeedy ſecretion of it, con-

" tain much ſaccharine matter. This is changed

" in ſome hours, by the animal proceſs, into

" an ammoniacal ſalt, which is that found in

" all the ſecretions. But the ſaccharine ſalt ſtill

" remaining in the urine, which is the moſt

" perfectly animaliſed fluid, ſhews that there is

" great defect in the animal proceſs *."

It muſt be allowed that ſugar is found — that ſweet chyle is the firſt product—and that urine is the moſt perfectly animaliſed fluid, &c. but it does not follow that the chyle is of no other uſe than to be converted into ammoniacal ſalt, or that any original defect in the animal proceſs is the cauſe of that want of converſion : this defect of ammoniacal ſalt, or rather ſaline matter, for the quantity of ammoniacal ſalt has been much over-rated, appearing, as I have attempted to explain above, to be owing to a want of phoſphoric acid, (which I take to be a modification of the ſaccharine) and the ſaccharine matter likewiſe appearing to be the cauſe rather than the effect of the diſeaſe.

* Home. Clinical Experiments, page 319.

I ſhall

I ſhall now farther obſerve, that the ſaline matter diſcharged by the kidnies in health ought to be conſidered as the product of nutrition, or rather the refuſe of that proceſs; for when the nutritive part of the blood has been applied to its various uſes, and ſecerned, are not the particles unfit for thoſe purpoſes retained, and brought back into the circulation to be diſcharged through the kidnies as excrementitious in various ſaline forms, which have been thus generated and rejected by the powers of nutrition?

May we not, in this manner, eaſily account for the ſmall quantity of excrementitious matter in the urine, without ſuppoſing any defect of aſſimilation? For where nutrition is very ſparingly performed, the quantity of excrementitious matter, the reſult of it, muſt be ſmall in proportion.

The remote or occaſional cauſes, noticed by authors, are, mineral and animal poiſons—intemperance in drinking and exerciſe — large doſes of antimonials — opiates and diuretics — large draughts, too frequently repeated, of Harrowgate and Epſom waters: to which may be added debility ſucceeding intermittents, and nephritic affections.

The method of cure likewiſe coincides with the idea of diabetes being a diſeaſe of the kidnies. Tonics, aſtringents, aromatics, agglutinants, abſorbents, and opiates, are the only medicines which have ſucceeded. Many cures performed by theſe, together with variety of formulæ, ſufficiently complicated and farraginous, may be found in the following works: — Vide Riolani op. p. 336. — Sennerti op. Tom. II. p. 1095. — Bonetus, lib. 4. — Pitcairn, p. 272. — Riverii op. p. 361. — Zacuti Luſitani op. p. 423. — Baglivi op. ep. 4. — R. Morton op. p. 15. — Martini Liſter Exercit. Med. p. 27. — Willis Ph. Rat. p. 105. — Etmulleri op. Tom. II. p. 714. — Hoffmanni Conſult. & Reſponſ. Medic. Caſus 85.

Upon the whole, conſidering the office of the kidnies to be merely that of percolation, I take the proximate cauſe of diabetes to conſiſt in a morbid dilatation of the uriniferous tubes of thoſe organs, whereby they become pervious to the nutritious matter, whoſe globuli, in a ſtate of health, are too large to be admitted through them; and that this morbid ſtate does exiſt either with or without a diarrhœa thereof.

When we conſider that the quantity of urine

 voided voided by Mr. Holford was singularly small, and that it did not contain latterly a greater proportion of saccharine matter than has been met with in other cases, where the patients have discharged four, five, or six times the quantity, and nevertheless withstood the ravages of the disease for years, the quantity of aliment demanded by the constitution and taken in having been adequate to the loss, is it not probable that a cure would have been effected, provided the stomach and organs subservient to digestion had retained their digestive power to supply the demands of the system?

*Chester,*

July 30, 1788.

V. *Obser-*

